# SARS-CoV-2/Renin–Angiotensin System: Deciphering the Clues for a Couple with Potentially Harmful Effects on Skeletal Muscle

**DOI:** 10.3390/ijms21217904

**Published:** 2020-10-24

**Authors:** Andrea Gonzalez, Josué Orozco-Aguilar, Oscar Achiardi, Felipe Simon, Claudio Cabello-Verrugio

**Affiliations:** 1 Laboratory of Muscle Pathology, Fragility and Aging, Department of Biological Sciences, Faculty of Life Sciences, Universidad Andres Bello, Santiago 8370146, Chile; a.gonzalezrojas@uandresbello.edu (A.G.); j.orozcoaguilar@uandresbello.edu (J.O.-A.); 2 Millennium Institute on Immunology and Immunotherapy, Santiago 8370146, Chile; fsimon@unab.cl; 3 Center for the Development of Nanoscience and Nanotechnology (CEDENNA), Universidad de Santiago de Chile, Santiago 8350709, Chile; 4 Escuela de Kinesiología, Facultad de Ciencias, Pontificia Universidad Católica de Valparaíso, Valparaíso 2340025, Chile; oscar.achiardi@pucv.cl; 5 Laboratory of Integrative Physiopathology, Department of Biological Sciences, Faculty of Life Sciences, Universidad Andres Bello, Santiago 8370146, Chile; 6 Millennium Nucleus of Ion Channels-Associated Diseases (MiNICAD), Universidad de Chile, Santiago 8370146, Chile

**Keywords:** COVID-19, SARS-CoV-2, muscle wasting, ICUAW, ICU

## Abstract

Severe acute respiratory syndrome coronavirus (SARS-CoV-2) has produced significant health emergencies worldwide, resulting in the declaration by the World Health Organization of the coronavirus disease 2019 (COVID-19) pandemic. Acute respiratory syndrome seems to be the most common manifestation of COVID-19. A high proportion of patients require intensive care unit admission and mechanical ventilation (MV) to survive. It has been well established that angiotensin-converting enzyme type 2 (ACE2) is the primary cellular receptor for SARS-CoV-2. ACE2 belongs to the renin–angiotensin system (RAS), composed of several peptides, such as angiotensin II (Ang II) and angiotensin (1-7) (Ang-(1-7)). Both peptides regulate muscle mass and function. It has been described that SARS-CoV-2 infection, by direct and indirect mechanisms, affects a broad range of organ systems. In the skeletal muscle, through unbalanced RAS activity, SARS-CoV-2 could induce severe consequences such as loss of muscle mass, strength, and physical function, which will delay and interfere with the recovery process of patients with COVID-19. This article discusses the relationship between RAS, SARS-CoV-2, skeletal muscle, and the potentially harmful consequences for skeletal muscle in patients currently infected with and recovering from COVID-19.

## 1. Introduction

Severe acute respiratory syndrome coronavirus (SARS-CoV-2) has been responsible for significant health emergencies worldwide since the end of 2019 and throughout 2020, leading to the coronavirus disease 2019 (COVID-19) pandemic. The World Health Organization reports 13,824,739 confirmed COVID-19 cases and 591,666 deaths worldwide until July 2020 [1]. This emergency makes it urgent to identify the mechanisms of action of the virus and the possible consequences. Finding the best therapeutic strategies to treat patients with SARS-CoV-2 as soon as possible, especially those in critical condition, is an essential step to prevent more deaths and complications for those who managed to survive.

The clinical characteristics of COVID-19 patients can range from an asymptomatic state to an upper airway infection to severe pneumonia associated with acute respiratory distress syndrome (ARDS), which requires ventilatory support [2,3,4,5,6]. Chest computed tomography images of patients with the virus have shown diffuse ground-glass opacities and early-stage lymphocytopenia even before dyspnea [7], indicating the severity of the disease. The clinical spectrum of pathology presents three main phases: early infection, pulmonary involvement, and systemic hyperinflammation. The symptoms are those of a respiratory infection—cough, fatigue, and shortness of breath—as well as less commonly systemic symptoms, such as headaches, myalgia, and arthralgia [4,7]. The risk factors for the increased severity of disease progression and increased death include comorbidities, such as high blood pressure, type 2 diabetes mellitus (T2DM), obesity, and cardiovascular disease (CVD), as well as an advanced age [2,5,6].

SARS-CoV-2 is a part of the β-coronavirus genus of the *Coronaviridae* family, to which SARS-CoV and Middle East respiratory syndrome coronavirus (MERS-CoV) also belong. Several members of this coronavirus family belong to α-coronavirus and β-coronavirus genera, which cause respiratory infections in humans [8,9,10,11]. The 30-kb genome of SARS-CoV-2 encodes a large auto-proteolytically non-structural protein that eventually forms the replicase–transcriptase complex. Moreover, the 3′ end of the viral genome encodes for four structural proteins, namely the spike (S), envelope (E), membrane (M), and nucleocapsid (N) proteins [9,12,13]. The SARS-CoV-2 genome shares a 79.6% sequence identity to SARS-CoV [10,14].

The crucial functional receptor for SARS-CoV-2 infection is angiotensin-converting enzyme 2 (ACE2), which belongs to the renin–angiotensin system (RAS) in humans, and it is highly expressed in the respiratory and intestinal tract [2,14,15,16,17]. SARS-CoV-2 receptor recognition is mediated by the glycosylated spike protein. After ACE2 binding, the S protein is cleaved and activated by transmembrane protease serine 2 into S1 and S2 subunits. S1 contains the receptor-binding domain, which directly binds to the peptidase domain (PD) of the ACE2 membrane, and the activated S2 subunit is responsible for membrane fusion [8,15,18,19]. Moreover, the receptor-binging domain in the S protein of SARS-CoV-2 differs in five of the six amino acid residues compared to SARS-CoV. These modifications probably explain the 10- to 20-fold higher affinity for ACE2 of SARS-CoV-2 compared with SARS-CoV [20,21].

ACE2 is part of the non-classical RAS axis [22]. ACE2 is a carboxypeptidase with two domains: a full extracellular amino-terminal PD domain and a carboxy-terminal collectrin-like domain containing a transmembrane helix intracellular segment [18,23]. The N-terminal catalytic domain of ACE2 produces angiotensin (1-7) (Ang-(1-7)) by two different processes, cleaving a residue from angiotensin I (Ang I) to produce angiotensin (1-9) (Ang-(1-9)), which has subsequent modifications made to it by other enzymes to become Ang-(1-7), and removing a single residue from angiotensin II (Ang II) to generate Ang-(1-7). Ang- (1-7) has a positive effect on different tissues because it promotes vasodilation, reduced proliferation, and prevents apoptosis [18,23].

ACE2 expresses in several tissues and organs in the body, such as the heart, kidney, small intestine, and, to a lesser extent, the lung and skeletal muscle [2,24]. It is highly expressed in the epithelium of the upper airway (nose and oropharynx), which is the principal entry point of SARS-CoV-2 in humans [7].

## 2. RAS Dysregulation and Its Relationship with COVID-19

RAS is a complex hormonal axis that, in physiological conditions, regulates blood pressure, hydro-electrolyte balance, inflammation, and fibrosis [25,26]. RAS is also one of the modulators of muscle mass [27,28]. RAS is divided into the following axes.

### 2.1. Classical Axis

**Figure 1:**
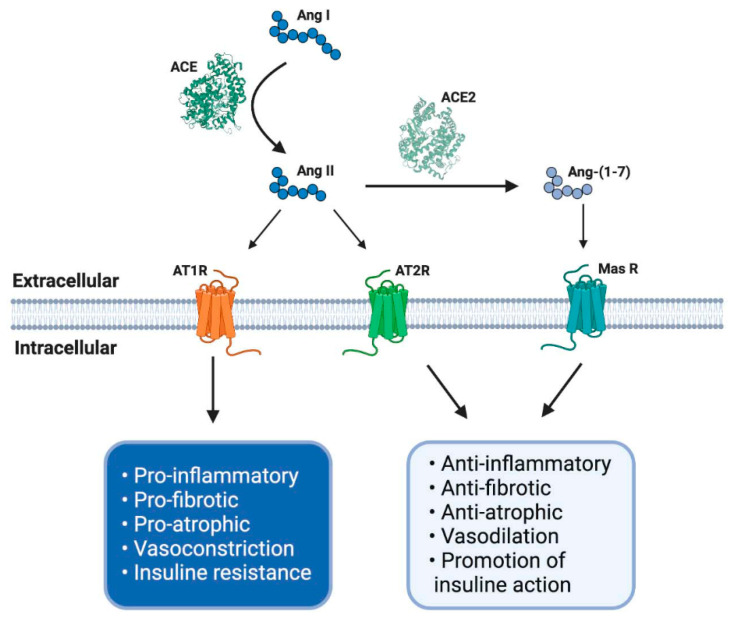
The renin–angiotensin system (RAS) and its physiological functions. The RAS regulates complex process as blood pressure, inflammation, carbohydrate metabolism or fibrosis, among others. It is composed of different peptides obtained by proteolytic cleavage mediated by specific enzymes belong to RAS. Thereby, angiotensin I (Ang I) is converted to Ang II by angiotensin-converting enzyme (ACE), and this second peptide can interact with its receptor angiotensin type 1 (AT1R), having some adverse biological effects, for example, an increase in blood pressure and pro-inflammatory events. However, Ang II, by its interaction with another receptor, AT2R, mediates opposite effects like vasodilatation and anti-inflammatory processes. Furthermore, Ang II can be converted to Ang-(1-7) by soluble ACE2 action and mediates the same beneficial effects through Mas receptor (MasR) signaling. ACE: angiotensin-converting enzyme; ACE2: angiotensin-converting enzyme 2; AT1R: angiotensin II type 1 receptor; AT2R: angiotensin II type 2 receptor; Ang I: angiotensin I; Ang II: angiotensin II; Ang-(1-7): angiotensin (1-7); MasR: Mas receptor; EC: extracellular; IC: intracellular. Created with BioRender.

This axis is composed of several peptides generated by the proteolytic action of enzymes belonging to RAS. Thus, Ang I is converted to Ang II by ACE. Ang II can bind to a family of G-protein-coupled receptors named angiotensin type 1 (AT1R) and type 2 (AT2R) receptors. The effects of AT1R-dependent Ang II and its intracellular signaling pathways result in harmful effects, such as inflammation, vasoconstriction, and atherogenesis, which can participate in the genesis of diseases, such as insulin resistance and thrombosis [29,30]. By contrast, AT2R stimulation by Ang II causes vasodilation, decreased platelet aggregation, and the promotion of insulin actions. Despite these beneficial effects, the expression of AT2R is low in most tissues in healthy adults [30] (Figure 1).

### 2.2. Non-Classical Axis

The effects of Ang II in adults are regulated and, in many cases, counteracted by the non-classical RAS axis [27,31,32]. In this axis, ACE2 converts Ang II to Ang-(1-7), which has beneficial effects, such as vasodilation and anti-fibrotic and anti-atrophic effects in skeletal muscle. Ang-(1-7) signals through the Mas receptor (MasR) and promotes similar biological effects as AT2R-mediated actions [26,33] (Figure 1).

**Figure 2:**
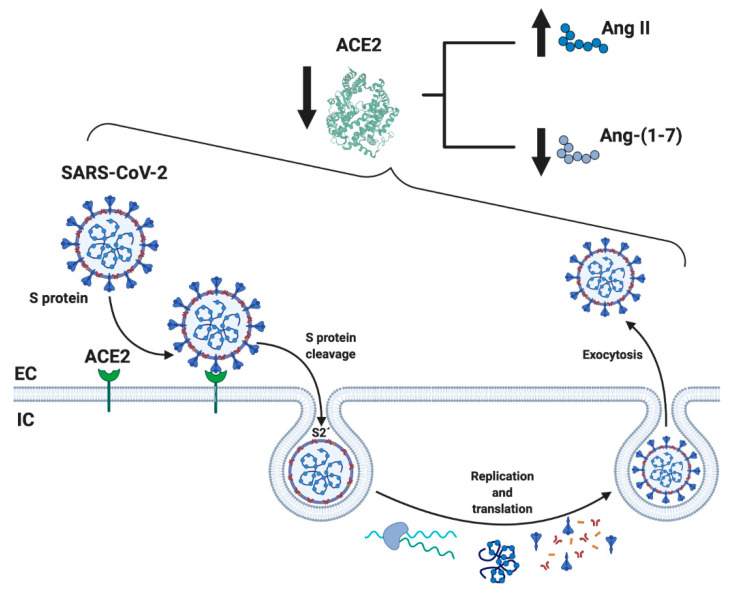
Schematic representation of the mechanism related to SARS-CoV-2 and RAS. SARS-CoV-2 binds through the spike (S) protein to its membrane receptor ACE2 in the respiratory epithelial membrane, permitting S protein’s cleavage by membrane proteases and exposing the S2’ fusion membrane domain to enter the cell by endocytosis and initiate the replication of the virus. One of the important consequences is the diminution of soluble ACE2 availability, resulting in subsequence increase and decrease levels of circulation Ang II and Ang-(1-7), respectively, causing a RAS imbalance. ACE2: angiotensin-converting enzyme 2; Ang-(1-7): angiotensin (1-7); EC: extracellular; IC: intracellular. Created with BioRender.

When SARS-CoV-2 enters human cells, it down-regulates the surface expression of ACE2 protein [34], which could occur due to the enzyme endocytosis complex with the virus protein S [7,35]. Furthermore, the binding of SARS-CoV-2 to ACE2 appears to induce ACE2 release as a soluble form in serum, further decreasing ACE2 activity [36,37]. These events would lead, on the one hand, to an exaggerated increase in the activation of the classical RAS pathway (ACE/Ang II/AT1R), which could induce a pro-fibrotic and pro-inflammatory state, vasoconstriction, increased membrane permeability, and apoptosis of lung epithelial cells [28,29,30,31] (Figure 2). This situation directly induces acute lung injury (ALI) and ARDS and can lead to death [10]. On the other hand, a decrease in the expression of ACE2 involves a reduction in Ang-(1-7), which could imply diminished anti-inflammatory, anti-fibrotic, and anti-atrophic effects. Both conditions, increases in Ang II/AT1R and decreases in ACE2/Ang-(1-7), have been identified in other chronic diseases, such as CVD and T2DM, and could happen in SARS-CoV-2 [18,25,38] (Figure 2).

This information would indicate that the dysregulation of RAS could be fundamental in the clinical development of SARS-CoV-2 [7]. Increased activity of ACE/Ang II/AT1R has been raised as a possible cause of the pathophysiological effects of SARS-CoV. This could produce an increase in the inflammatory and fibrotic state and a decrease in ACE2/Ang-(1-7)/MasR activity, which would also happen in SARS-CoV-2 [39,40].

In this regard, it has been demonstrated that ACE2 decreases its expression in mice with severe ALI induced by acid aspiration or sepsis. Simultaneously, components of the classical RAS pathway (ACE, Ang II, AT1R) increase at the pulmonary and systemic levels. These changes promote the pathogenesis of lung disease, induce edemas, and impair lung function. The authors conclude that ACE2 has a protective effect in mice with ALI [39]. Furthermore, it has been demonstrated that the SARS-CoV spike protein increases Ang II and ACE2 down-regulation, resulting in lung injury [34]. ACE2 is upregulated through a negative feedback mechanism by blocking AT1R, leading to lung protection from virus damage, which could be attributed to the increased conversion of Ang II to Ang-(1-7) [41]. Furthermore, it has been found that ACE2 down-regulation induces the persistent elevation of Ang II through local interaction with the AT1R, triggering a vicious cycle in which Ang II down-regulates ACE2, leading to a local increase in Ang II in the tissues [42].

To date, the use of ACE and AT1R blockers (ARB) as a possible treatment to reduce lung inflammatory response and mortality in patients with COVID-19 pneumonia could confirm that RAS dysregulation is a part of the pathophysiology of COVID-19. However, this is still controversial because, for example, ARB can increase ACE2 expression, causing harmful consequences for patients with COVID-19 [43,44].

## 3. RAS and Its Role in the Loss of Muscle Mass

Skeletal muscle is the most abundant tissue in mammals, and it has critical functions in body homeostasis, such as movement, body support, breathing, heat generation, and endocrine function. Thus, muscle mass loss alters the proper functioning of the body.

A loss of muscle mass can occur because of various causes, such as aging, malnutrition, prolonged rest, physical inactivity, and chronic diseases (neurological, cardiac, respiratory, endocrine, etc.) [45]. A decrease in muscle mass is associated with other negative consequences, such as loss of strength and physical performance, a syndrome known as sarcopenia [46].

Sarcopenia is a crucial determinant of frailty, leading to a loss of autonomy and functionality in daily living activities, hospitalization, and the institutionalization of patients [47]. Sarcopenia mechanisms include increased protein degradation and decreased synthesis, autophagy dysregulation, increased oxidative stress and myonuclear apoptosis, and mitochondrial dysfunction [28,48]. Soluble molecules regulate these mechanisms; these are called atrophic factors, and one of them is Ang II [28,49,50].

All the components of RAS are found in skeletal muscle. Therefore, dysregulation of both axes will directly affect skeletal muscle mass and function [27]. In this regard, classical RAS pathway activation has been associated with detrimental consequences in skeletal muscle, such as muscular atrophy, fibrosis, and insulin resistance [24,27]. Ang II binding to AT1R can generate a cascade of intracellular events in skeletal muscle, including increased reactive oxygen species (ROS) production, protein degradation, development of fibrosis, and decreased protein synthesis [27,32]. Increased Ang II levels have been observed in chronic pathologies, such as heart failure, chronic kidney failure, and obesity. This event has significant clinical relevance because sarcopenia occurs secondary to most chronic diseases, and it can accelerate the loss of functionality and increase morbidity and mortality [27].

As mentioned before, the canonical RAS pathway’s increased activation induces protein degradation and decreases synthesis in skeletal muscle, which regulates muscle mass [51]. An increase in circulating Ang II stimulates Ser-307 phospho-insulin receptor substrate 1, which alters the protein kinase B (Akt)/mammalian target of rapamycin (mTOR) signaling pathway and reduces the levels of insulin-like growth factor type 1 (IGF1) and thus muscle protein synthesis. Because of the aforementioned, Ang II also blocks insulin signaling in skeletal muscle, affecting the translocation of glucose transporter type 4 (GLUT-4) to the sarcolemma and glucose homeostasis [27,52]. Furthermore, Ang II can induce the activation of caspase-3 and the dephosphorylation of Akt, which allows the nuclear translocation of FoxO1 to increase the expression of two muscle-specific E3 ligases belonging to the ubiquitin–proteasome system (UPS), atrogin-1/muscle atrophy F-box, and muscle RING-finger protein-1 (MuRF-1) [53,54]. Thus, mechanisms that impair the insulin-IGF1/mTOR signaling pathway and UPS overactivation contribute to skeletal muscle mass loss in almost all atrophic conditions [27,53]. On the other hand, the Ang-(1-7)/MasR signaling pathway’s activation prevents harmful effects dependent on Ang II in skeletal muscle and, therefore, prevents sarcopenia [31,55].

Ang II/AT1R increases inflammation, an event associated with increased muscle wasting in chronic pathologies. This inflammatory process could occur through the activation of NF-κB due to the phosphorylation and ubiquitination of IκBα protein in a ROS-dependent way. This effect could be increased by pro-inflammatory cytokines, such as interleukins [56]. In this context, IL-6 would act by enhancing UPS activation and E3-ligase expression (catabolic path), decreasing the levels of IRS-1 and phosphorylated Akt (anabolic path), and increasing the suppressor of cytokine signaling 3 expression (inflammatory path) [57]. Ang II can also activate the tumoral necrosis factor α (TNF-α) /TNF receptor 1 complex, which inactivates Akt, favoring glycogen synthase kinase 3 beta (GSK3β) activation, and, consequently, the activation of UPS and protein degradation [58]. Finally, Ang II/AT1R binding can activate transforming growth factor β (TGFβ) signaling, leading to an inflammatory state through extracellular signal-regulated kinase 1/2 and Jun N-terminal kinase pathway activation [59]. Ang-(1-7)/MasR axis shows opposite effects on TGFβ signaling, decreasing TGFβ expression in skeletal muscle [60].

As a counterpart, the activation of the non-classical RAS pathway produces Ang-(1-7) production through the degradation of Ang II-mediated by ACE2. Ang-(1-7), through MasR, regulates and counteracts many of the negative actions of Ang II/AT1R [22,27,31,32]. ACE2, meanwhile, inactivates Ang II and is a negative regulator of Ang II-dependent signaling [39]. In this regard, ACE2/Ang-(1-7)/MasR in skeletal muscle shows anti-atrophic, anti-fibrotic, and anti-inflammatory activities [27,31,32,61,62,63]. In murine models, this axis can increase the muscle strength and functionality of animals [64], and it can also increase the activity of Akt/mTOR-p70S6K, favoring the synthesis of muscle proteins. Furthermore, non-classical RAS axis activation can decrease muscle protein degradation by inducing several events, such as decreased activity and expression of MuRF-1 and atrogin-1, diminution of ROS production, and prevention of nuclear factor-kappa beta (NF-κB) signaling activation [24,27,31,32,62,65]. Ang-(1-7) can also prevent a decrease in muscle fibers’ diameter and avoid the transition in their type [31,62,66].

## 4. Dysregulation of RAS by COVID-19 and Its Possible Harmful Effects on Skeletal Muscle

Skeletal muscle can be severely affected by RAS dysregulation as a result of chronic pathologies. As mentioned previously, the mechanisms involved reduce the synthesis and increase muscle protein degradation as an inflammatory and fibrotic process. These events lead to muscle mass loss, strength, and physical function, affecting people’s quality of life. If a period of hospitalization (e.g., in the intensive care unit (ICU)) or invasive mechanical ventilation (IMV) and sepsis are added to this chronic condition, the deterioration of skeletal muscle can be much more severe and aggressive, and recovery could be prolonged.

In SARS-CoV-2, there is a hyperinflammatory state with a dysregulation of RAS that could induce atrophy of skeletal muscle with all the associated functional consequences. If the condition is severe, it may cause ARDS, shock, myocardial injury, acute kidney injury, and multi-organ failure, aggravating the state and probably leading to ICU admission and IMV use [67]. The latter is a form of invasive support used in severe forms of COVID-19. The prolonged use of IMV on ICU patients is directly associated with further complications, such as ventilator-associated pneumonia, pneumothorax and pneumomediastinum, and ventilator-induced diaphragmatic dysfunction (VIDD), leading to elevated mortality [68].

In a recent study with 73 adult patients (median age: 61 years, mostly male (83.6%)), COVID-19 ARDS was associated with prolonged mechanical ventilation (MV) and high short-term mortality, among other factors [67]. In another study involving 21 cases (average age: 70 years old, 52% male), all patients developed severe ARDS, 81% were admitted to the ICU, most of them needed MV (71%), and they had a high mortality rate (67%) [69].

The severe inflammation observed in SARS-CoV-2 patients could increase the classical RAS axis and decrease the expression and activity of the non-classical axis. These events, added to the patients’ ICU status and MV, could generate a harmful effect in skeletal muscle that could slow down recovery or increase death risk.

In this context, muscle wasting involving diaphragmatic and lower limb muscles is experienced by 50% of ICU patients, causing severe respiratory and physical complications that might remain for years after hospital discharge [70]. Considering other pathologies similar to COVID-19, a dysfunction in the diaphragm muscle has been seen as a characteristic of severe symptoms, so it is expected that this behavior could be identical in COVID-19 [68,71,72].

## 5. Diaphragmatic Dysfunction for Invasive Mechanical Ventilation in COVID-19 Patients

Considering all antecedents and the clinical outcomes in patients with COVID-19, there is a high probability that patients in the ICU, especially those with IMV, can develop diaphragmatic dysfunction.

Diaphragmatic dysfunction is one of the most significant consequences of using IMV. From a physiological perspective, diaphragm dysfunction is directly related to diaphragm weakness (DW). It can be defined as the diaphragm’s reduced ability to generate a negative intrathoracic pressure, usually less than 11 cm H_2_O [73,74]. The prevalence of diaphragmatic dysfunction in critically ill patients who require intubation, such as those affected by COVID-19, is high at 60%. It can be as high as 80% in patients requiring prolonged MV and experiencing difficult weaning [75]. DW also correlates with higher mortality, and it is a more reliable predictor of ICU mortality [75].

DW in patients with IMV may be caused by the underlying effects of pathologies, sepsis, and other systemic infections that are responsible for many cases of ICU-acquired DW. Other factors that contribute to DW are the drugs used during ICU stay (neuromuscular blocking agents and corticosteroids) and atrophy resulting from IMV use [73,75].

In humans, the use of IMV causes weaning failures in approximately 20% of patients because of the rapid deterioration of diaphragm muscle endurance and strength; this condition is called ventilator-induced diaphragmatic dysfunction (VIDD) [76]. VIDD is the most significant factor for failed weaning in mechanically ventilated patients [73]. The mechanisms underlying VIDD could be the following: (1) disuse atrophy secondary to diaphragm inactivity from excessive ventilatory support [77]; (2) hypercapnia, which induces a marked reduction in diaphragm force as assessed by phrenic nerve stimulation [78]; and (3) excessive loading, which causes structural damage and myofiber remodeling (load-induced injury because of insufficient ventilatory support) [73,79].

Diaphragm dysfunction is associated with pathophysiological changes in skeletal muscle, which are common in both animal and human studies, and include increased oxidative stress, muscle fiber atrophy, and injury and the activation of several major proteolytic pathways (ubiquitin–proteasome, caspases, calpains) [80,81,82,83]. In VIDD, muscle atrophy in the diaphragm with decreased type II muscle fibers (fast-twitch) within the early course of the disease has been reported (12 to 18 h after controlled mechanical ventilation [CMV] [81,84]. There is also a remodeling process with an increase in hybrid fibers concomitantly with a decrease in type I fibers (slow twitch), which can be identified at later stages [85]. Together with these changes, observing abnormal sarcomere structure areas and an irregular Z-line structure in the diaphragm is possible [83].

Protein synthesis pathways are decreased in VIDD [86]. Six hours of CMV is associated with a 30% decrease in mixed protein synthesis and a 65% decline in myosin heavy-chain protein synthesis [86]. These changes remained consistent throughout 18 h after CMV [83]. Furthermore, proteolytic systems are activated in animal models of VIDD and also in patients with MV. Specifically, there is an activation of the calpain and caspase systems, UPS, and the autophagy–lysosomal systems [73,79,84,87,88,89].

Redox disturbance also occurs because of prolonged CMV, which results in increased ROS production and diminished antioxidant capacity in the diaphragm [83,90]. This redox alteration has adverse effects on crucial contractile proteins, such as actin and myosin, which are oxidized in the diaphragm during prolonged CMV and lipid peroxidation [91]. These events have high relevance because redox disturbances in skeletal muscle promote contractile dysfunction and the activation of proteolytic systems [82,83]. Finally, CMV produces extensive diaphragm remodeling through the alteration of gene expression. In this condition, stress-sensitive genes are upregulated, whereas structural protein and energy metabolism genes are down-regulated from 6 to 18 h after CMV [83,92].

The contribution of the classical RAS axis to DW has been reported in a preclinical model of MV. In this model, there are high circulating levels of Ang II. The relevance of this fact is that with the use of AT1R blockers, DW was recovered, suggesting that Ang II participates in the generation of DW [93]. The mechanisms involved in Ang II-dependent DW were previously discussed in this review. Interestingly, there is evidence showing the preventive effect of the non-classical RAS axis on MV-induced DW. Thus, Ang-(1-7) administration during MV has a protective role on the diaphragm’s muscular fibers, maintaining muscular fiber features, and reducing atrophy [66].

Although there is no known history of the presence of diaphragmatic dysfunction in patients with COVID-19 in the ICU and MV to date, it is most likely that it is occurring, as the alteration is indifferent to the cause of MV. However, it could even worse because of the loss of muscle mass associated with RAS dysregulation mentioned earlier.

## 6. ICU-Acquired Weakness in COVID-19

Another possible consequence of ICU stays and the use of MV in COVID-19 patients may be the development of ICU-acquired weakness (ICUAW).

ICUAW is a limb and respiratory weakness syndrome that develops in the wake of critical illness [94]. The incidence of ICUAW is approximately 80% in ICU patients, and it is associated with a longer duration of MV and hospitalization, along with more significant functional impairment for survivors [95]. ICUAW is a manifestation of nerve and muscle dysfunction due to generalized systemic inflammation and risk factors such as sepsis, shock, and the presence of multi-organ failure [96]. Although some risk factors are present in COVID-19 patients, ICUAW has yet to be determined in those critically ill with SARS-CoV-2.

ICUAW commonly manifests in three different manners, which can often coincide: polyneuropathy, myopathy, and muscle atrophy [97].

Critical illness polyneuropathy (CIP) and critical illness myopathy (CIM) are characterized by flaccid and symmetric paralysis, producing limb and respiratory, skeletal muscle weakness. However, in CIP, there is a distal axonal polyneuropathy in sensory and motor nerves. Meanwhile, in CIM, the sensory function is preserved despite muscle weakness [95,98]. The etiology for the development of CIP and CIM is not fully understood. Despite this, it could include microcirculatory abnormality (loss of the blood–nerve barrier), metabolic or bioenergetic dysfunction, channelopathy (inexcitability of the endoneural or muscle membrane), and/or direct toxic effects of ICU care (hyperglycemia or lipids in parenteral nutrition in CIP and corticosteroids or neuromuscular blockade in CIM) [98].

Muscle atrophy was observed in type II fiber together with myosinolysis (proteolytic degradation of myosin), which is consistent with primary myopathy and neurogenic muscle atrophy [95,97].

These pathophysiological disorders translate clinically into a loss of strength and muscle mass, weakness, and significant functional disorders in daily living activities, aside from being an independent predictor of mortality in critically ill patients [95].

Patients with ICUAW maintained significantly worse handgrip strength and reported worse physical functioning-related quality of life [99]. Despite improvements in overall strength in the timeline, physical function-related quality of life remained significantly below the expected age-adjusted indicators at all-time points [99].

In the case of COVID-19, to date, there have been no studies on ICUAW. There is limited research indicating the medium-term characteristics of recovered COVID-19 patients with or without the MV requirement. An analysis performed in three hospitals in Wuhan, China, showed that critically ill patients requiring ICU admission and MV, or oxygen therapy, had a considerable 60-day mortality. Furthermore, an age older than 65 years, thrombocytopenia at ICU admission, ARDS, and acute kidney injury were independent predictors of these patients’ 60-day mortality [100]. Similar results were recently reported, and they showed that the survival time of non-survivors is likely to be within 1–2 weeks after ICU admission [101].

There is a report that tracked individuals who were discharged from the hospital after recovery from COVID-19. This study shows that the mean length of a hospital stay was 13.5 days. However, the most critical data were those on the evaluation of patients 60 days after the onset of the first COVID-19 symptom; at the time of the assessment, only 12.6% were completely free of any COVID-19–related symptoms, 32% had one or two symptoms, and 55% had three or more. The four most frequent symptoms at 60 days after the onset of the first COVID-19 symptoms were fatigue (53.1%), dyspnea (43.4%), joint pain (27.3%), and chest pain (21.7%) [102].

These are the first studies to identify the medium-term effects of the virus, the risk of death, and the disabling symptoms. Among the latter, the most prevalent is fatigue, which could be related to the loss of muscle mass associated with RAS dysregulation that we discussed previously and would be even more aggressive in ICU and IMV patients. A significant complication in ICU patients is weakness and muscle mass loss, including that of appendicular and respiratory muscles, such as the diaphragm. A percentage of patients with COVID-19 require ventilatory support and an ICU stay, which can undoubtedly affect their muscle mass. The reasons for this can be the use of MV and prolonged rest and the dysregulation of the RAS axis, the latter of which is a part of the disease’s pathophysiology.

Therefore, studying and analyzing ICUAW and its phases in patients with COVID-19 are necessary. Despite the importance of exploring the preventive and treatment aspects of COVID-19 patients, particular emphasis must be placed on these patients’ recovery phase. Antecedents that support this fact indicate that the loss of muscle mass, strength, and physical performance will be one of the main limitations of recovery from ICUAW.

**Figure 3:**
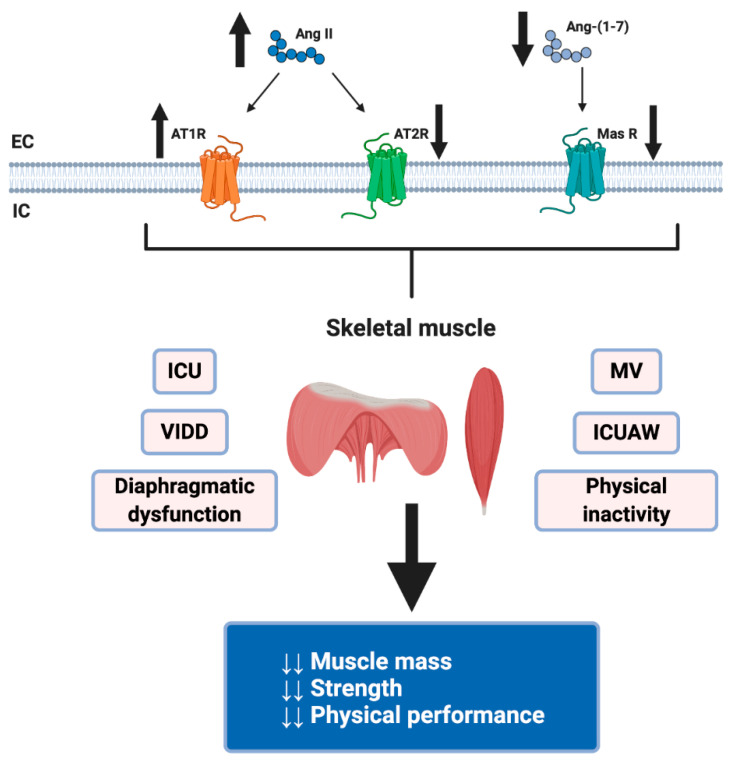
Highlights the possible mechanism by SARS-CoV-2 induces skeletal muscle atrophy through RAS dysregulation. RAS dysregulation due to SARS-CoV2 infection (decreased Ang II and Ang-(1-7)) plus its hospitalization consequences, for example, mechanical ventilation (MV) use, intensive care unit (ICU) stays, ventilator-induced diaphragmatic dysfunction (VIDD), ICU-acquired weakness (ICUAW), diaphragmatic dysfunction in critical patients and physical inactivity due to public health recommendations in sustained quarantine, could originate loss of skeletal muscle mass and strength and decreased physical performance. Created with BioRender.

The side effects related to infection by SARS-CoV-2 negatively influence skeletal muscle health [103]. These events are related to immobilization because of hospitalization and bed rest, IMV use, ICU stay, and physical inactivity due to public health recommendations for sustained quarantine (remaining at home, closing parks, gyms, and fitness centers) to prevent the spread of SARS-CoV-2. These pathological consequences of COVID-19 could be attributed partly to the deregulation of RAS (Figure 3).

## 7. Possible Therapeutic Interventions

Potential therapies to treat skeletal muscle complications due to SARS-CoV-2 are yet to be investigated. Most research is still focused on the early stages of the pathology and because it has been a relatively short time since its outbreak. Based on the well-established treatments for skeletal muscle health recovery in other similar conditions, it is possible to speculate that therapeutic interventions should include pharmacological and non-pharmacological approaches [104].

The pharmacological approach could consider blocking the classical RAS pathway and/or increasing the activity of the non-classical pathway and anti-inflammatory drugs. Regarding the classical RAS pathway, the ACE inhibitors and angiotensin receptor blockers (ARBs) could be considered a good alternative. Nevertheless, it has been hypothesized that they could increase Ang II plasma levels, increasing ACE2 expression, and inducing more target molecules to be available for the SARS-CoV-2 virus [105,106,107]. At present, no evidence has shown that continued use of ACE inhibitors and ARBs increases the risk of SARS-CoV-2 severe infection or the risk of death [108,109,110,111]. At skeletal muscle levels, a minor activity of the RAS classical pathway could decrease the atrophic stimulus.

On the other hand, it is known that Ang-(1-7)/MasR axis induces skeletal muscle protein synthesis and decreases its degradation. Ang-(1-7)/MasR increases the activity of Akt/mTOR-p70S6K, favoring the synthesis of muscle proteins and reducing protein degradation, by decreasing ROS, IKK, NF-κB and the activity of MuRF-1 and atrogin-1 [24,27,31,32,62,65]. Moreover, Ang-(1-7) can prevent a decrease in muscle fiber diameter and avoid the transition in their type [31,62,66]. This non-classical RAS action could decrease the negative SARS-CoV-2 consequences in the skeletal muscle through Ang-(1-7) effects.

The most widely used drugs for treating acute inflammation in COVID-19 are corticosteroids, but they can directly induce muscle atrophy and weakness [112]. Immunotherapies, such as IL-1 and IL-6 inhibitors, which do not have the adverse effects of corticosteroids on skeletal muscle, are also being investigated to treat acute inflammation in patients with COVID-19 [113].

As for the non-pharmacological approach, the primary therapeutic tool to treat the musculoskeletal condition is exercise. Based on the proposed skeletal muscle consequences of RAS deregulation, exercise interventions should focus on two main muscle groups: respiratory muscles and appendicular muscles. The pulmonary function of patients surviving COVID-19 pneumonia is affected, with a restrictive pattern even 6 weeks after hospital discharge [114]. A recent study with a 6-week respiratory rehabilitation program, which included diaphragmatic and respiratory muscle training and cough exercises, showed an improvement in pulmonary function and 6-min walk distance [115]. However, further clinical trials are still needed to identify the optimal training parameters for respiratory rehabilitation in COVID-19 survivors.

Exercise training is well known to increase skeletal mass, strength, and physical performance [116]. Unfortunately, to date, there is no published evidence on physical training programs, whether aerobic, strength or combined, that focus on skeletal muscle health in COVID-19 patients. In this regard, resistance training could be a primary therapeutic tool to rectify SARS-CoV-2 consequences, especially when there is a low tolerance to the effort, as occurs in other chronic respiratory patients [117,118,119]. In people with a greater tolerance for exercising, endurance training could improve physical performance, dyspnea, and fatigue symptoms [117,119]. Furthermore, evidence indicates that endurance training has been shown to induce an increase in the non-classical RAS pathway, which elevates Ang-(1-7) levels with the positive effects mentioned above [120,121].

## 8. Conclusions

Considering this background, we propose that skeletal muscle tissue is severely affected by SARS-CoV-2. Among the mechanisms involved in this dysfunction are the increased activity of Ang II/AT1R and the lower activity of ACE2 and Ang-(1-7). This unbalanced RAS activity, together with IMV use, ICU stay, prolonged rest, and a lack of physical activity, leads to the loss of muscle mass, strength, and physical function. All these factors have adverse effects on patients’ recovery toward their total functional level and may even influence post-virus mortality. Appropriate and timely evaluation and treatment of skeletal muscle disease are essential to allow patients to recover more quickly from functional limitations. Further research is needed on potential therapies that focus on the loss of muscle mass, strength, and physical function in COVID-19 patients.

## Abbreviations

ACE	angiotensin-converting enzyme
ACE2	angiotensin-converting enzyme 2
Akt	protein kinase B
ALI	acute lung injury
Ang I	angiotensin I
Ang II	angiotensin II
Ang-(1-7)	angiotensin (1-7)
Ang-(1-9)	angiotensin (1-9)
ARB	angiotensin II type 1 receptor blocker
ARDS	acute respiratory distress syndrome
AT1R	angiotensin II type 1 receptor
AT2R	angiotensin II type 2 receptor
CIM	critical illness myopathy
CIP	Critical illness polyneuropathy
CMV	controlled mechanical ventilation
COVID-19	coronavirus disease 2019
CVD	cardiovascular disease
DW	diaphragm weakness
ICU	intensive care unit
ICUAW	ICU-acquired weakness
IGF1	insulin-like growth factor type 1
IL-6	Interleukin 6
IMV	invasive mechanical ventilation
MasR	Mas receptor
mTOR	mammalian target of rapamycin
MuRF-1	muscle RING-finger protein-1
NF-κβ	Nuclear factor-kappa beta
PD	peptidase domain
RAS	renin–angiotensin system
ROS	reactive oxygen species
SARS-CoV	Severe acute respiratory syndrome coronavirus
SARS-CoV-2	Severe acute respiratory syndrome coronavirus 2
T2DM	Type 2 diabetes mellitus
TGFβ	transforming growth factor β
UPS	ubiquitin–proteasome system
VIDD	ventilator-induced diaphragmatic dysfunction
